# Apical myocardial fibrosis burden identifies a high-risk phenotype and predicts cardiac mortality after LVAD implantation

**DOI:** 10.1093/eschf/xvag135

**Published:** 2026-05-12

**Authors:** İbrahim Demir, Bilge Ecemiş, Ayşe Zorba, Işık İkbal Barış, Berhan Keskin, Emir Cantürk, Yahya Yıldız, İbrahim Oğuz Karaca, Korhan Erkanlı

**Affiliations:** Department of Cardiovascular Surgery, Istanbul Medipol University, Istanbul 34214, Turkey; Department of Cardiovascular Surgery, Istanbul Medipol University, Istanbul 34214, Turkey; Department of Cardiovascular Surgery, Istanbul Medipol University, Istanbul 34214, Turkey; Department of Pathology, Istanbul Medipol University, Istanbul 34214, Turkey; Department of Cardiology, Istanbul Medipol University, Istanbul 34214, Turkey; Department of Cardiovascular Surgery, Istanbul Medipol University, Istanbul 34214, Turkey; Department of Anesthesiology and Reanimation, Istanbul Medipol University, Istanbul 34214, Turkey; Department of Cardiology, Istanbul Medipol University, Istanbul 34214, Turkey; Department of Cardiovascular Surgery, Istanbul Medipol University, Istanbul 34214, Turkey

**Keywords:** Myocardial fibrosis, Histopathology, Heart failure, Left ventricular assist device

## Abstract

**Introduction:**

Advanced heart failure is characterized by progressive myocardial remodelling, with fibrosis representing a final common pathway of chronic injury. We investigated whether quantitatively assessed apical myocardial fibrosis burden at the time of left ventricular assist device (LVAD) implantation is associated with overall and cause-specific mortality.

**Methods:**

This single-centre retrospective cohort study included 47 consecutive patients undergoing durable LVAD implantation between 2019 and 2025. Median age was 63 years and 14.9% were female. Myocardial tissue obtained from the left ventricular apical core at implantation was analysed using structured histopathologic scoring (THS-10) and quantitative digital morphometry (QuPath). Fibrosis burden was quantified as collagen-positive area relative to total myocardial area. Overall survival was assessed using Kaplan–Meier analysis and Cox regression. Cardiac mortality was evaluated using Fine–Gray competing-risk models, with non-cardiac death treated as a competing event.

**Results:**

Median follow-up was 1513 days. During follow-up, 27 deaths occurred (57.4%), including 16 cardiovascular and 11 non-cardiac deaths. Quantitative fibrosis burden showed a strong unadjusted association with overall survival (log-rank *P* = .00042). Receiver operating characteristic (ROC) analysis identified a cohort-derived fibrosis threshold of 33.7% for overall mortality risk stratification (area under the curve [AUC] 0.739, 95% confidence interval [CI] 0.566–0.912). In univariable Cox analysis, fibrosis burden and THS-10 score were associated with mortality (hazard ratio [HR] 39.39, 95% CI 2.61–594.56, *P* = .008; and HR 1.51, 95% CI 1.14–2.01, *P* = .005, respectively). After multivariable adjustment for clinical severity, including INTERMACS profile and key laboratory parameters, the association with all-cause mortality was attenuated. In competing-risk analysis, high fibrosis burden independently predicted cardiac mortality (subdistribution HR approximately 4.1, *P* = .02). Additional exploratory ROC analysis for cardiovascular death showed an AUC of 0.752, with a cohort-derived threshold of 39.6%.

**Conclusion:**

Quantitatively assessed apical myocardial fibrosis burden identifies a biologically high-risk myocardial phenotype in LVAD recipients. While the association with all-cause mortality is attenuated after adjustment, fibrosis burden shows a specific relationship with cardiac mortality when competing non-cardiac risk is taken into account. These findings support the prognostic relevance of myocardial substrate and warrant external validation in larger prospective cohorts.

## Graphical Abstract

**Graphical Abstract:**
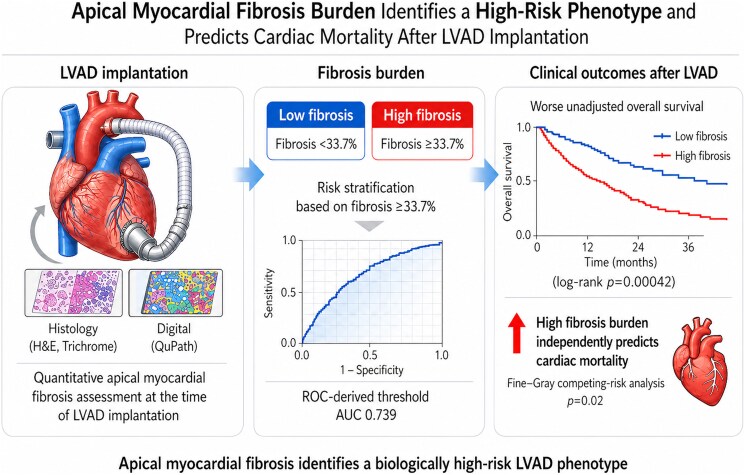

## Introduction

Advanced heart failure is characterized by progressive structural remodelling of the myocardium, with fibrosis representing the final common pathway of chronic injury, neurohormonal activation, and inflammatory signalling.^1,2^ Fibrotic expansion disrupts myocardial architecture, increases ventricular stiffness, impairs contractile efficiency, and promotes malignant arrhythmogenesis, thereby exerting a profound influence on clinical outcomes.^3,4^

Left ventricular assist devices have transformed the management of end-stage heart failure by restoring systemic perfusion and unloading the failing ventricle. However, clinical outcomes after left ventricular assist device (LVAD) implantation remain heterogeneous, suggesting that pre-implant myocardial substrate plays a critical role in determining long-term prognosis.^5,6^ Prior studies have demonstrated variable degrees of reverse remodelling following LVAD support, yet persistent fibrosis frequently limits myocardial recovery and predisposes to adverse outcomes.^5,7^

Despite growing recognition of the importance of myocardial fibrosis, most prior LVAD studies relied on qualitative histology or indirect imaging surrogates, limiting their ability to define clinically actionable thresholds.^8,9^ Moreover, the prognostic relevance of fibrosis quantified using digital morphometry and integrated with histopathologic features has not been systematically evaluated.

In this context, we sought to determine whether quantitatively assessed myocardial fibrosis burden predicts long-term survival and cause-specific mortality following LVAD implantation.

## Methods

This single-centre retrospective cohort study included all consecutive patients who underwent durable LVAD implantation between January 2019 and December 2025. Clinical, echocardiographic, laboratory, and outcome data were retrieved from institutional electronic medical records, national mortality registries, and standardized outpatient follow-up. The patients’ baseline laboratory and echocardiographic parameters were monitored during admission. Cardiac catheterizations were performed 24–48 h prior to surgery during the same admission process. Eligible patients had implantation of a HeartMate II or HeartMate III device, availability of intraoperative apical myocardial tissue, and complete survival follow-up. Patients lacking adequate tissue samples or digital histopathologic imaging were excluded.

### Histopathologic processing and semi-quantitative scoring

Apical core samples were obtained exclusively at the time of LVAD implantation as part of the standard coring procedure. Importantly, no post-implant biopsies, RV biopsies, or postmortem tissues were used, ensuring a homogeneous sampling strategy and preventing artefacts related to ischaemic time or postmortem degradation.^1,3^ Tissues were fixed in 10% neutral buffered formalin, embedded in paraffin, and archived in the institutional pathology repository.

Semi-quantitative histologic assessment was performed using the Total Histopathology Score (THS-10), which integrates four core myocardial injury domains: inflammatory infiltrates (INFL), interstitial fibrosis (IS-F), perivascular fibrosis (PV-F), and replacement myocardial scarring (SCAR), each graded according to predefined severity scales (*Table 1*).

**Table 1:** Semi-quantitative and quantitative histopathologic scoring system used for myocardial tissue assessment

Component	Parameter	Scale	Criteria
THS-10	INFL—Inflammation (H&E)	0—4	0: None 1: Perivascular inflammatory infiltrate 2: Interstitial infiltrate 3: Interstitial infiltrate + focal myocyte loss 4: Interstitial infiltrate + confluent myocyte loss
	IS-F—Interstitial Fibrosis (PSR)	0—3	0: None 1: Limited (scattered, small area) 2: Moderate (widespread but not diffuse) 3: Diffuse increase
	PV-F—Perivascular fibrosis (PSR)	0—2	0: None 1: <50% vascular circumference with fibrosis 2: ≥50% vascular circumference with fibrosis
	SCAR—Myocardial scar (H&E/PSR)	0—1	0: Absent 1: Present (single or multiple; clinically relevant)
	Total THS-10 Score	0—10	Sum of INFL + IS-F + PV-F + SCAR
	Severity categories	—	0—2 Minimal/None 3—5 Mild 6—8 Moderate 9—10 Severe
QAS	Inflammation (% area)	0—2	0: <0.5% 1: 0.5—5% 2: >5%
	Fibrosis (% area)	0—2	0: <1% 1: 1—5% 2: >5%
	Total QAS Score	0—4	Inflammation% bin (0–2) + Fibrosis% bin (0–2)
Integrated score	THS-10 + QAS	0—14	Combined semiquantitative-digital morphometry score

In parallel, a Quantitative Area Score (QAS) was derived from digital morphometric analysis of histologic sections stained with Picrosirius red and haematoxylin–eosin. Fibrotic and inflammatory tissue burden was quantified as the percentage of collagen-positive and inflammatory area relative to total myocardial area, subsequently categorized into ordinal bins and summed to generate a total QAS (range 0–4).

**Figure 1:**
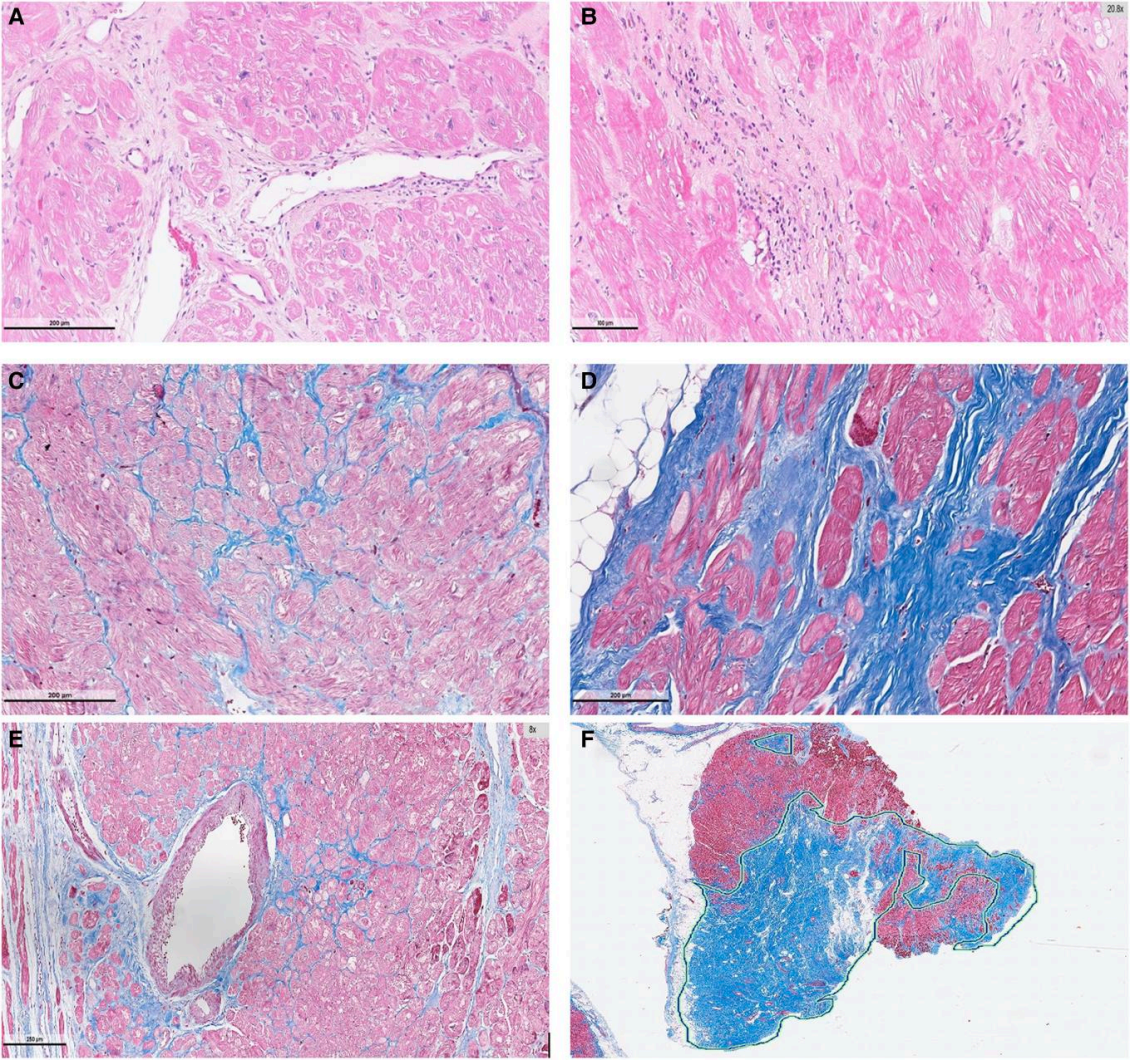
Histopathological assessment and quantitative analysis of myocardial fibrosis. Representative histological sections obtained from LV apical core biopsies at the time of LVAD implantation. (*A*) Perivascular inflammatory cell infiltration corresponding to inflammation score 1 (haematoxylin and eosin [H&E] staining). (*B*) Interstitial inflammatory infiltrate with focal myocyte loss corresponding to inflammation score 3 (H&E staining). (*C*) Mild interstitial fibrosis corresponding to fibrosis score 1 (Masson’s trichrome staining). (*D*) Diffuse interstitial fibrosis corresponding to fibrosis score 3 (Masson’s trichrome staining). (*E*) Perivascular fibrosis highlighted by Masson’s trichrome staining. (*F*) Quantitative digital morphometric analysis demonstrating extensive myocardial fibrosis (fibrosis burden 66%) performed using QuPath software; fibrotic tissue is highlighted as blue in all figures

An integrated myocardial injury score was then calculated by combining the THS-10 and QAS components, yielding a composite semi-quantitative–digital score that captures both qualitative histopathologic severity and objective fibrosis burden (*Table 1*). Representative stained myocardial sections illustrating the spectrum of fibrosis and inflammatory involvement are shown in *Figure 1*.

### Statistical analysis

Continuous variables are presented as medians with interquartile ranges, and categorical variables as counts and percentages. Overall survival was defined as the time from LVAD implantation to death from any cause.

The prognostic associations of demographic, clinical, laboratory, echocardiographic, haemodynamic, histopathologic, and quantitative morphometric variables were first assessed using univariable Cox proportional hazards regression. INTERMACS profile was prespecified and analysed as INTERMACS 1–2 vs. 3–7 to reflect high-risk versus more stable clinical status at implantation. Underlying cardiomyopathy was classified as ischaemic or non-ischaemic according to the documented coronary artery disease status in the database, which corresponded to ischaemic cardiomyopathy categorization in our cohort.

Myocardial fibrosis was evaluated using complementary approaches. Quantitative fibrosis burden derived from digital morphometry was analysed as a continuous variable, and a data-derived threshold for overall mortality was determined using receiver operating characteristic (ROC) curve analysis with the Youden index. Discrimination was quantified using the area under the curve (AUC) with corresponding confidence intervals (CIs). Based on this cut-off, patients were classified as having low or high fibrosis burden for subsequent analyses.

Multivariable models were constructed in a parsimonious manner because of the limited sample size and event count. Variable selection was based on a combination of clinical relevance, avoidance of collinearity, and the need to limit model complexity, rather than on univariable significance alone. INTERMACS profile, albumin, and creatinine were prioritized as clinically relevant indicators of illness severity at implantation. Although age was significantly associated with mortality in univariable analysis, it was not included in the primary multivariable models to avoid overparameterization in this small cohort.

To assess the independent prognostic contributions of clinical severity and myocardial substrate while minimizing collinearity, three prespecified multivariable Cox models were constructed. Proportional hazards assumptions were verified using Schoenfeld residuals. Overall survival was illustrated using Kaplan–Meier curves and compared by the log-rank test. Given the exploratory nature of the study and the limited sample size, no formal adjustment for multiple testing was applied to the univariable analyses. These results should therefore be interpreted as hypothesis generating rather than confirmatory.

Cause-specific mortality was analysed using Fine–Gray competing-risk regression, with cardiac death as the event of interest and non-cardiac death as the competing event; cumulative incidence functions were generated accordingly. In additional exploratory analyses, cardiovascular mortality was also examined using cause-specific Cox regression, with non-cardiac death treated as a censoring event. ROC analysis was repeated using cardiovascular death as the endpoint.

All tests were two-sided, with *P* < .05 considered statistically significant. Analyses were conducted using R software (R Foundation for Statistical Computing, Vienna, Austria).

## Results

### Cohort characteristics and fibrosis distribution

A total of 47 consecutive patients undergoing surgery were included. The median age was 63 years (interquartile range: 54–70.5). Baseline echocardiography demonstrated uniformly severe systolic dysfunction, with a median left ventricular ejection fraction of 20%.

Quantitative digital morphometry–based fibrosis measurements were available in 36 patients. Using ROC analysis, a data-derived fibrosis threshold of 33.7% was identified for risk stratification within the present cohort, and this cut-off was used to categorize patients into high vs. low fibrosis burden groups for downstream survival and competing-risk analyses.

During follow-up, a total of 27 deaths occurred. Cause-specific adjudication identified 16 cardiac/LVAD-related deaths and 11 non-cardiac deaths. LVAD-related deaths were attributable to right ventricular failure (*n* = 12), intracranial haemorrhage (*n* = 3), and device thrombosis (*n* = 1). Non-cardiac deaths were due to sepsis (*n* = 1), COVID-19–related complications (*n* = 5), intestinal bleeding (*n* = 4), and motor-vehicle accident (*n* = 1). Baseline demographic, clinical, laboratory, echocardiographic, and haemodynamic characteristics are summarized in *Table 2*.

**Table 2:** Baseline characteristics

Variable	Fibrosis rate <33.7%	Fibrosis rate ≥33.7%	*P-value*
	*n* = 22	*n* = 14	
Sex			*>*.*9*
Male	18 (82%)	12 (86%)	
Female	4 (18%)	2 (14%)	
Age	60 (52–70)	68 (59–73)	.*079*
BMI (kg/m^2^)	28.7 (24.9–31.5)	28.7 (26.0–33.7)	.*5*
NYHA			.*7*
2	1 (4.5%)	0 (0%)	
3	2 (9.1%)	0 (0%)	
4	19 (86%)	14 (100%)	
INTERMACS profile			**.*004***
1	0 (0%)	4 (29%)	
2	1 (4.5%)	2 (14%)	
3	9 (41%)	1 (7.1%)	
4	6 (27%)	7 (50%)	
5	4 (18%)	0 (0%)	
6	1 (4.5%)	0 (0%)	
7	1 (4.5%)	0 (0%)	
Underlying cardiomyopathy			.*67*
Ischaemic cardiomyopathy	16 (73%)	12 (86%)	
Non-ischaemic cardiomyopathy	6 (27%)	2 (14%)	
Concomitant diseases			
Hypertension	19 (86%)	12 (86%)	*>*.*9*
Diabetes mellitus	9 (41%)	9 (64%)	.*2*
Chronic kidney disease	10 (45%)	10 (71%)	.*13*
Cerebrovascular event	0 (0%)	2 (14%)	.*14*
Atrial fibrillation	6 (27%)	7 (50%)	.*2*
Coronary artery disease	16 (73%)	12 (86%)	.*4*
Pacemaker implantation	20 (91%)	11 (79%)	.*4*
Medications			
ARNI usage	9 (41%)	3 (21%)	.*292*
ACEi/ARB usage	6 (27%)	4 (29%)	*>*.*9*
SGLT-2 inhibitor usage	7 (32%)	6 (43%)	.*723*
Diuretic usage	16 (73%)	12 (86%)	.*441*
Beta-blocker usage	22 (100%)	11 (79%)	.*051*
MRA usage	9 (41%)	6 (43%)	*>*.*9*
Blood tests			
Haemoglobin (g/dL)	11.45 (9.90–12.90)	10.45 (8.70–14.10)	.*9*
WBC (x10^9^/L)	7.4 (6.3–9.6)	9.7 (7.6–13.6)	.*067*
Platelets (x10^4^/L)	176 (144–239)	229 (193–284)	.*2*
Creatinine (mg/dL)	1.14 (.91–1.80)	1.95 (1.54–3.51)	**.*002***
Albumin (g/dL)	3.92 (3.52–4.35)	3.48 (2.84–3.71)	**.*006***
CRP (mg/L)	21 (11–53)	42 (11–68)	.*5*
NT-proBNP (pg/mL)	4819 (1736–8233)	5890 (3476–8115)	.*6*
Cardiac measurements			
LVEF (%)			.*030*
10	1 (4.5%)	1 (7.1%)	
15	8 (36%)	0 (0%)	
20	11 (50%)	11 (79%)	
25	2 (9.1%)	2 (14%)	
TAPSE/PASP (mm/mmHg)	0.32 (0.23–0.43)	0.28 (0.25–0.36)	.*3*
Cardiac Output (L/min)	3.86 (3.44–4.61)	3.40 (3.02–4.11)	.*3*
Cardiac Index (L/min/m^2^)	1.90 (1.76–2.22)	1.70 (1.52–2.18)	.*3*
PCWP (mmHg)	24 (16–30)	30 (25–35)	.*2*
PAPM (mmHg)	25 (21–38)	36 (27–40)	.*083*
PVR (Woods Unit)			.*2*
0	4 (21%)	2 (15%)	
1	9 (47%)	6 (46%)	
2	2 (11%)	1 (7.7%)	
3	3 (16%)	0 (0%)	
4	0 (0%)	3 (23%)	
6	1 (5.3%)	0 (0%)	
7	0 (0%)	1 (7.7%)	
PAPi	2.85 (2.30–3.23)	3.30 (1.69–3.57)	.*4*

Quantitative fibrosis burden was numerically higher in the ICM group than in the nICM group (0.314 [0.213–0.398] vs. 0.188 [0.070–0.302], *P* = .148). Similarly, THS-10 scores were numerically higher in patients with ICM (6.5 [5.0–8.0] vs. 5.5 [3.0–6.5], *P* = .281). The proportion of patients with high fibrosis burden according to the ROC-derived cut-off was also greater in the ICM group (42.9% vs. 25.0%, *P* = .441), although these differences did not reach statistical significance.

### Survival analyses and predictors of mortality

In univariable Cox regression analyses, advanced clinical severity at implantation was strongly associated with overall mortality compared with INTERMACS 3–7 (*P* < .001). Increasing age was also a significant predictor (*P* < .001). Histopathological markers of myocardial injury were robustly associated with mortality. Both the THS-10 score (*P* = .005) and quantitative myocardial fibrosis rate (*P* = .008) predicted adverse outcomes. Higher serum creatinine was associated with increased mortality (*P* = .005), whereas higher serum albumin was protective (*P* = .002). Other clinical, echocardiographic, and haemodynamic variables were not significantly associated with overall mortality (*Table 3*).

**Table 3:** Univariable cox proportional hazards regression for overall mortality

Variables		HR (95% CI)	*P-*value
Clinical characteristics			
INTERMACS Profile	*INTERMACS 3–7*	Ref	
	*INTERMACS 1–2 (High Risk)*	6.31 (2.58–15.43)	**<.001**
NYHA profile (per class increase)		3.01 (0.49–18.25)	.232
Age		1.08 (1.04–1.13)	**<.001**
Sex (female)		0.59 (0.20–1.75)	.346
BMI (kg/m^2^)		1.00 (0.93–1.07)	.903
Pre-existing diabetes mellitus		0.91 (0.41–2.01)	.817
Pre-existing coronary artery disease		1.69 (0.63–4.52)	.297
Pre-existing hypertension		1.07 (0.37–3.14)	.901
Pre-existing atrial fibrillation		1.11 (0.49–2.50)	.810
Pre-existing cerebrovascular event		4.20 (0.93–19.05)	.063
Pre-existing chronic kidney disease		1.28 (0.59–2.78)	.529
Presence of ICD		0.49 (0.21–1.11)	.088
Medications			
ARNI usage		0.58 (0.24–1.39)	.221
ACEi/ARB usage		0.69 (0.29–1.65)	.399
SGLT-2 inhibitor usage		0.66 (0.28–1.53)	.330
Diuretic usage		0.94 (0.35–2.51)	.900
Beta-blocker usage		0.44 (0.16–1.19)	.107
MRA usage		0.50 (0.21–1.20)	.121
Histopathological scoring			
THS-10 score^1–10^		1.51 (1.14–2.01)	**.005**
Fibrosis rate (per %1 increase)		39.39 (2.61–594.56)	**.008**
Laboratory parameters			
Creatinine (mg/dL)		1.56 (1.14–2.12)	**.005**
Albumin (g/dL)		0.43 (0.25–0.73)	**.002**
Haemoglobin (mg/dL)		0.85 (0.69–1.03)	.100
NT-proBNP (logarithmic)		1.00 (1.00–1.00)	.133
Echocardiographic and catheterization measurements			
LVEF (%)		1.09 (0.96–1.25)	.190
Cardiac index (L/min/m^2^)		2.20 (0.95–5.08)	.064
Cardiac output (L/min)		1.21 (0.80–1.81)	.369
TAPSE/PASP (mm/mmHg)		0.27 (0.01–14.57)	.523
PAPD (mmHg)		0.98 (0.93–1.04)	.554
PCWP (mmHg)		1.01 (0.96–1.07)	.673
PAPM (mmHg)		0.99 (0.95–1.03)	.674
PVR (Woods unit)		0.96 (0.76–1.23)	.764
PAPi (per 1 increase)		0.96 (0.71–1.29)	.771

To address potential confounding and collinearity, three prespecified multivariable Cox models were constructed. In Model 1, INTERMACS 1–2 remained independently associated with mortality (*P* = .032), whereas fibrosis rate showed a non-significant trend.

In Model 2, substituting fibrosis rate with the THS-10 score, both THS-10 score (*P* = .040) and INTERMACS 1–2 (*P* = .004) remained independently associated with mortality.

In Model 3, using ROC-defined high fibrosis burden as a categorical variable, INTERMACS 1–2 persisted as an independent predictor (*P* = .022), while high fibrosis burden was no longer significant after adjustment (*Table 4*).

**Table 4:** Multivariable cox proportional hazards regression models for overall mortality

Variables	HR (95% CI), *P- value*	HR (95% CI), *P- value*	HR (95% CI), *P- value*
Model 1			
Albumin (g/dL)	0.42 (0.21–0.85), ***.015***	—	—
Creatinine (mg/dL)	1.00 (0.69–1.44), .998	—	—
Fibrosis ratio (%)	12.20 (0.34–443.37), .172	—	—
INTERMACS 1–2 (high-risk)	3.93 (1.13–13.72), ***.032***	—	—
Model 2			
Albumin (g/dL)	—	0.48 (0.25–0.94), ***.033***	—
Creatinine (mg/dL)	—	1.07 (0.75–1.51), .724	—
THS-10 score	—	1.38 (1.01–1.87), ***.040***	—
INTERMACS 1–2 (high-risk)	—	5.14 (1.69–15.65), ***.004***	—
Model 3			
Albumin (g/dL)	—	—	0.51 (0.25–1.03), .060
Creatinine (mg/dL)	—	—	1.01 (0.71–1.45), .944
INTERMACS 1–2 (high-risk)	—	—	4.33 (1.24–15.15), ***.022***
Fibrosis rate (High vs. Low)	—	—	2.01 (0.55–7.43), .294

### Kaplan–Meier survival analysis according to fibrosis burden

**Figure 2:**
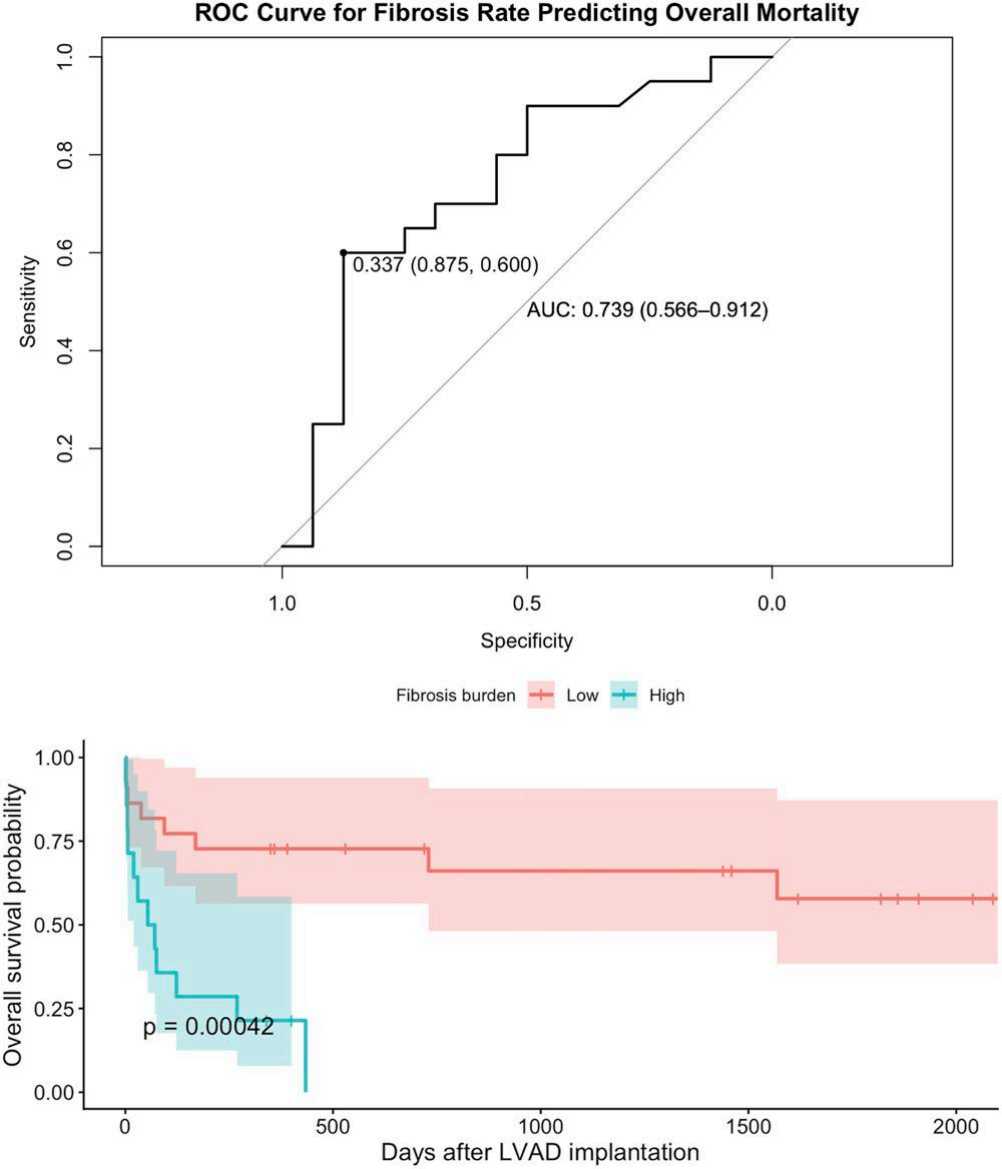
The survival curves separate early after implantation and remain widely divergent throughout follow-up, indicating a strong unadjusted association between myocardial fibrosis burden and long-term mortality

Kaplan–Meier analysis demonstrated a marked separation of survival curves according to myocardial fibrosis burden stratified by the ROC-derived cut-off. Patients with high fibrosis burden exhibited significantly worse overall survival (*log-rank P* = .00042) (*Figure 2*). Survival divergence occurred early after LVAD implantation and persisted throughout long-term follow-up.

### Competing risk analysis for cardiac mortality (Fine–Gray model)

**Figure 3:**
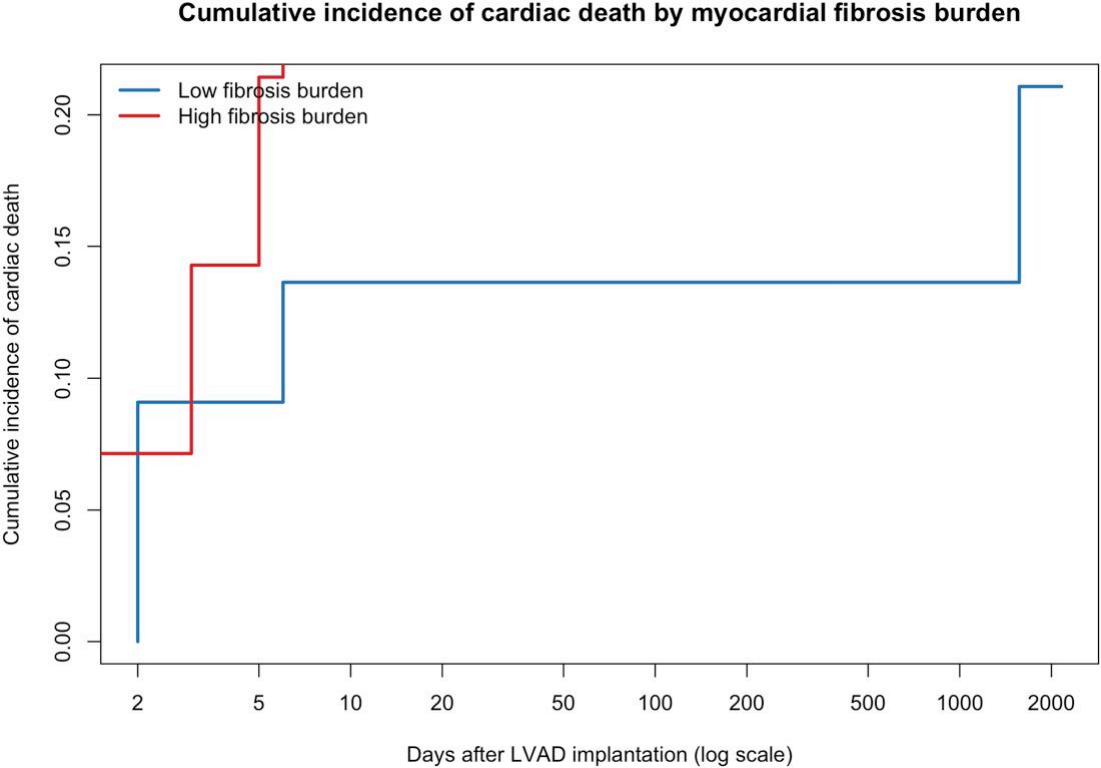
Cumulative incidence of cardiac death according to myocardial fibrosis burden. Cardiovascular mortality was presented as a cumulative incidence function rather than a Kaplan–Meier curve because non-cardiac death was treated as a competing event

Given the high proportion of non-cardiac deaths, a competing risk analysis was performed to assess the relationship between myocardial fibrosis burden and cardiac mortality. Fine–Gray regression demonstrated that high fibrosis (33.7%) was independently associated with an increased cumulative incidence of cardiac death (*P* = .02). Cumulative incidence curves showed early and persistent separation in the high-fibrosis group throughout follow-up (*Figure 3*).

As an additional exploratory analysis, cardiovascular mortality was further examined as a cause-specific endpoint. In ROC analysis, quantitative fibrosis burden showed good discrimination for cardiovascular death, with an AUC of 0.752 and a data-derived threshold of 39.6% (Supplementary Figure S2). In cause-specific univariable Cox analysis, higher fibrosis burden was significantly associated with cardiovascular mortality. In multivariable analyses, this association remained directionally consistent but was attenuated after adjustment, whereas INTERMACS 1–2 remained the strongest independent predictor. These findings were concordant with the competing-risk analysis and further support the biological relevance of fibrosis burden for cardiac mortality (Supplementary Table S2). Exploratory spline-based Cox modelling of quantitative fibrosis burden suggested a directionally increasing association with overall mortality across the observed range, although CIs widened at the extremes because of the limited number of observations (Supplementary Figure S3).

### Device subgroup analyses

Exploratory subgroup analyses were performed according to LVAD device type to assess the consistency of the association between myocardial fibrosis burden and overall survival. In both HeartMate II and HeartMate III subgroups, patients with high fibrosis burden demonstrated worse survival (Supplementary Figure S1A and B). Given the limited sample size, these analyses were considered descriptive and hypothesis generating.

## Discussion

In this study, we demonstrate that quantitatively assessed myocardial fibrosis burden at the time of LVAD implantation is strongly associated with long-term outcomes, with differential implications depending on the mortality endpoint. While higher fibrosis burden is associated with markedly worse unadjusted survival, its prognostic relevance becomes most apparent for cardiac mortality when non-cardiac death is explicitly accounted for as a competing risk. Fibrosis burden was numerically higher in patients with ischaemic cardiomyopathy, although this difference was not statistically significant, likely reflecting limited power in this relatively small cohort. These findings suggest that myocardial fibrosis reflects a biologically vulnerable myocardial substrate rather than serving as a sole causal driver of adverse outcomes.

### Myocardial fibrosis as a marker of advanced structural remodelling

Myocardial fibrosis reflects the cumulative effects of chronic myocardial injury driven by pressure and volume overload, neurohormonal activation, inflammation, and microvascular dysfunction in advanced heart failure.^1,3^ Both interstitial and replacement fibrosis have been linked to impaired compliance, electrical instability, and progressive ventricular dysfunction.^2,3^ In the setting of LVAD support, such structural alterations may persist despite mechanical unloading, limiting myocardial adaptability.

Our findings extend prior histopathological observations by providing quantitative evidence that the degree of fibrotic remodelling at implantation carries prognostic information beyond conventional clinical and echocardiographic parameters. The wide variability in fibrosis burden observed—despite uniformly advanced heart failure—highlights substantial biological heterogeneity among LVAD candidates, consistent with previous tissue-based studies.^5,11^

### Differential impact on all-cause and cardiac mortality

The attenuation of the association between myocardial fibrosis burden and all-cause mortality after multivariable adjustment likely reflects the complex mortality landscape of contemporary LVAD recipients. This divergence reflects the complex mortality profile of long-term LVAD recipients, in whom non-cardiac causes such as infection, bleeding, and systemic complications substantially contribute to overall mortality.^6,8,12^

By contrast, competing-risk analysis isolates cardiac mortality as a mechanistically relevant outcome, revealing a robust association between fibrosis burden and cardiac death. This approach aligns with contemporary methodological recommendations for advanced heart failure research, where competing risks are common and traditional Cox models may underestimate substrate–outcome relationships.^9^ Additional cause-specific analyses for cardiovascular mortality yielded results consistent with the competing-risk framework, further supporting the biological specificity of fibrosis burden for cardiac rather than systemic modes of death.

### Interaction between fibrosis burden and clinical severity

INTERMACS profile remained the strongest independent predictor of mortality across multivariable models, underscoring its role as an integrative marker of clinical instability at implantation. Nevertheless, myocardial fibrosis burden consistently demonstrated directional associations with adverse outcomes after adjustment for INTERMACS and other clinical variables. This suggests that fibrosis burden captures a complementary biological dimension—irreversible myocardial structural damage—not fully encompassed by clinical severity indices alone.

The observed association between higher fibrosis burden and impaired right ventricular–pulmonary arterial coupling further supports the concept of ventricular interdependence in advanced heart failure.^7^ Extensive left ventricular fibrosis may adversely affect septal mechanics and limit right ventricular adaptation following LVAD implantation, thereby predisposing patients to early haemodynamic compromise.

Myocardial fibrosis represents the cumulative consequence of chronic myocardial injury driven by neurohormonal activation, inflammation, microvascular dysfunction, and mechanical stress. In the setting of LVAD support, extensive interstitial and replacement fibrosis may limit myocardial adaptability to unloading, impair ventricular compliance, and predispose to electrical instability and right ventricular–pulmonary arterial uncoupling. Accordingly, fibrosis burden may identify patients in whom mechanical support cannot fully mitigate underlying myocardial vulnerability.

### Device subgroup analyses and platform consistency

Exploratory analyses demonstrated consistent directional associations between fibrosis burden and survival across LVAD platforms. Although underpowered for formal interaction testing, these findings suggest that myocardial substrate may continue to influence outcomes despite advances in device technology. Larger multicentre studies will be required to determine whether fibrosis burden modifies device-specific outcomes.^12,13^

### Clinical perspectives

Despite similar clinical indications for LVAD implantation, patients exhibit substantial biological heterogeneity. Our findings identify myocardial fibrosis burden as a tissue-level marker of disease irreversibility that provides prognostic information beyond established clinical staging systems such as INTERMACS. Unlike haemodynamic or laboratory parameters, fibrosis reflects the cumulative burden of chronic myocardial injury and maladaptive remodelling.^2,3^

The persistence of a strong association between fibrosis burden and cardiac mortality in a competing risk framework underscores its biological specificity.^14^ These findings support prior histopathological observations that advanced interstitial and replacement fibrosis may limit myocardial resilience under mechanical unloading,^5,11^ highlighting the importance of integrating myocardial substrate into LVAD risk assessment.

### Strengths and limitations

Several limitations warrant consideration. The single-centre design and moderate sample size limit extensive multivariable adjustment and generalizability. Nevertheless, this constraint reflects the inherent challenges of tissue-based LVAD research and was addressed through parsimonious modelling and complementary competing-risk analyses. Myocardial sampling was restricted to apical core tissue and may not capture regional heterogeneity; however, prior LVAD tissue studies suggest that apical specimens can provide a representative window into global myocardial remodelling in advanced heart failure.^1–3^ The definition of ischaemic cardiomyopathy based on documented coronary artery disease may not fully distinguish true ischaemic cardiomyopathy from bystander coronary artery disease in patients with non-ischaemic cardiomyopathy. In addition, multiple univariable comparisons were performed without formal correction for multiplicity; accordingly, these findings should be interpreted with caution and considered exploratory. The ROC-derived fibrosis threshold was generated within the present cohort and may be sample specific; therefore, prospective external validation in larger independent multicentre cohorts is required before broader clinical application. Finally, causality cannot be inferred from this observational design.

### Conclusion

Taken together, our findings support the concept that myocardial fibrosis burden represents a tissue-level marker of irreversible myocardial vulnerability in patients undergoing LVAD implantation. By specifically associating fibrosis burden with cardiac mortality within a competing-risk framework, this study highlights the importance of myocardial substrate in shaping long-term outcomes beyond conventional clinical staging in advanced heart failure.

## Supplementary Material

xvag135_Supplementary_Data

## Declarations

### Data Availability

The datasets generated and analysed during the current study are not publicly available due to patient privacy restrictions but are available from the corresponding author on reasonable request and pending institutional approval.

### Pre-registered Clinical Trial Number

None supplied.
