# Supplementary material for: Apical myocardial fibrosis burden identifies a high-risk phenotype and predicts cardiac mortality after LVAD implantation
Source: ESC Heart Fail. 2026 May 12;13(3):xvag135. doi: 10.1093/eschf/xvag135 (PMC13195508; doi:10.1093/eschf/xvag135)
Supplement: xvag135_Supplementary_Data [file xvag135_supplementary_data.zip › Supplementary Table S2.docx]

**Supplementary Table S2. Cause-specific cardiovascular mortality analyses**

*Additional exploratory analyses with cardiovascular death as the endpoint*

**Part A. Univariable cause-specific Cox regression for cardiovascular mortality**

| **Variable** | **HR (95% CI)** | **p-value** |
| --- | --- | --- |
| INTERMACS 1–2 (high-risk) | 11.73 (3.15–43.72) | **<0.001** |
| Age | 1.09 (1.02–1.16) | **0.012** |
| Albumin (g/dL) | 0.41 (0.19–0.85) | **0.017** |
| Creatinine (mg/dL) | 1.20 (0.77–1.87) | 0.414 |
| THS-10 score | 1.30 (0.94–1.81) | 0.118 |
| Fibrosis rate (%) | 121.14 (3.73–3929.87) | **0.0069** |

**Part B. Multivariable cause-specific Cox regression models for cardiovascular mortality**

| **Variable** | **Model 1** | **Model 2** | **Model 3** |
| --- | --- | --- | --- |
| Albumin (g/dL) | 0.41 (0.15–1.12), p=0.081 | 0.48 (0.18–1.31), p=0.151 | 0.54 (0.19–1.53), p=0.244 |
| Creatinine (mg/dL) | 0.75 (0.45–1.26), p=0.278 | 0.82 (0.50–1.34), p=0.423 | 0.80 (0.49–1.31), p=0.369 |
| Fibrosis rate (%) | 61.97 (0.53–7294.35), p=0.090 | – | – |
| THS-10 score | – | 1.14 (0.81–1.61), p=0.452 | – |
| High fibrosis burden  (≥33.7) | – | – | 2.02 (0.33–12.40), p=0.448 |
| INTERMACS 1–2 (high-risk) | 5.06 (1.22–20.92), **p=0.025** | 8.45 (2.16–32.99), **p=0.002** | 6.63 (1.37–31.98), **p=0.019** |

Abbreviations: HR, hazard ratio. Model 1 included albumin, creatinine, fibrosis rate, and INTERMACS 1–2. Model 2 included albumin, creatinine, THS-10 score, and INTERMACS 1–2. Model 3 included albumin, creatinine, high fibrosis burden, and INTERMACS 1–2. These analyses were performed as additional exploratory analyses using cardiovascular death as the outcome and non-cardiac death as a censoring event.
