# Supplementary material for: Apical myocardial fibrosis burden identifies a high-risk phenotype and predicts cardiac mortality after LVAD implantation
Source: ESC Heart Fail. 2026 May 12;13(3):xvag135. doi: 10.1093/eschf/xvag135 (PMC13195508; doi:10.1093/eschf/xvag135)
Supplement: xvag135_Supplementary_Data [file xvag135_supplementary_data.zip › Supplementary-Highlights and Graphical Abstract.docx]

**Highlights**

• Quantitative digital morphometry reveals marked interpatient heterogeneity in myocardial fibrosis burden at the time of LVAD implantation.

• Higher myocardial fibrosis burden is associated with significantly worse unadjusted long-term survival and early divergence of Kaplan–Meier survival curves.

• While the association with all-cause mortality is attenuated after adjustment for clinical severity, competing-risk analysis demonstrates that high fibrosis burden independently predicts cardiac mortality.

• These findings identify myocardial fibrosis as a tissue-level marker of irreversible myocardial vulnerability that complements conventional clinical risk stratification in advanced heart failure.

**Graphical Abstract Narrative**

This graphical abstract illustrates the prognostic relevance of myocardial fibrosis burden in patients undergoing left ventricular assist device (LVAD) implantation. Myocardial tissue obtained from the left ventricular apical core at the time of implantation was analysed using integrated histopathologic scoring and quantitative digital morphometry, revealing substantial interpatient heterogeneity in fibrosis burden despite uniformly advanced heart failure.

Patients with higher myocardial fibrosis burden demonstrated markedly worse unadjusted long-term survival, with early and persistent separation of Kaplan–Meier curves following LVAD implantation. Receiver operating characteristic analysis identified a clinically meaningful fibrosis threshold that stratified patients into distinct risk groups.

Given the substantial contribution of non-cardiac causes to mortality in LVAD recipients, a competing-risk framework was applied. When non-cardiac death was treated as a competing event, high myocardial fibrosis burden emerged as a specific and independent predictor of cardiac mortality, highlighting a biologically relevant association between myocardial substrate and cardiac vulnerability under mechanical unloading.

Collectively, the graphical abstract emphasizes that myocardial fibrosis burden reflects irreversible myocardial remodeling and identifies a biologically high-risk LVAD phenotype, complementing conventional clinical severity indices and refining tissue-level risk stratification in advanced heart failure.
