# Supplementary material for: Apical myocardial fibrosis burden identifies a high-risk phenotype and predicts cardiac mortality after LVAD implantation
Source: ESC Heart Fail. 2026 May 12;13(3):xvag135. doi: 10.1093/eschf/xvag135 (PMC13195508; doi:10.1093/eschf/xvag135)
Supplement: xvag135_Supplementary_Data [file xvag135_supplementary_data.zip › Supplementary-Histopathology Methods.docx]

**H&E Examination:** Hematoxylin–eosin (H&E) stained sections were reviewed for:
• inflammatory infiltrates (location and severity),
• myocyte injury and dropout,
• interstitial expansion, and
• architectural disarray.

**Masson Trichrome Staining:** Fresh 3–4 µm sections were stained with Masson Trichrome to characterize interstitial fibrosis, perivascular collagen accumulation, and replacement scar formation. Myocardial fibers stained red, whereas collagen and fibrosis stained blue, allowing clear discrimination of fibrotic burden and pattern.

**THS-10 Histopathology Score**: A structured semi-quantitative scoring system (THS-10) was applied, adapted from prior myocardial fibrosis literature and calibrated for LVAD tissue morphology (3,4,10). Each case received a total score (0–10) based on the following components:

• **INFL**: inflammatory activity (0–4)
• **IS-F**: interstitial fibrosis (0–3)
• **PV-F**: perivascular fibrosis (0–2)
• **SCAR**: replacement scarring (0–1)

Detailed definitions for each score bin are shown in **Table 1** and were applied consistently to all specimens. All histopathologic evaluations were performed by an experienced cardiovascular pathologist (blinded to clinical outcomes). Inter-observer reproducibility was ensured by a second independent reading of 25% of specimens; disagreements were resolved by consensus.

**Digital Fibrosis Quantification (QuPath Workflow)**

Whole-slide images (WSI) were digitized at ×20 magnification using a Hamamatsu NanoZoomer scanner and exported in high-resolution NDPI format. Digital analysis was conducted in QuPath (v0.4+), following a standardized pipeline:

1. Region of interest annotation of all myocardial areas (excluding pericardial fat, hemorrhage, artifacts).
2. Color deconvolution with Masson Trichrome presets.
3. Automated pixel-classifier separation of collagen (blue) vs myocardium (red).
4. Calculation of fibrosis ratio = collagen-positive area / total myocardial area (continuous variable).

Digital fibrosis values were used in all quantitative analyses. Whole-slide images were analyzed using QuPath. Collagen-positive area divided by total myocardial area yielded the fibrosis ratio (5,8). The ROC-Youden cutoff (%33.7) stratified high vs low fibrosis burden. A supplementary Quantitative Area Score (QAS, 0–4) was also computed, integrating percent-area inflammation and fibrosis as defined in **Table 1**.
