# Supplementary figures and images for: Apical myocardial fibrosis burden identifies a high-risk phenotype and predicts cardiac mortality after LVAD implantation
Source: ESC Heart Fail. 2026 May 12;13(3):xvag135. doi: 10.1093/eschf/xvag135 (PMC13195508; doi:10.1093/eschf/xvag135)

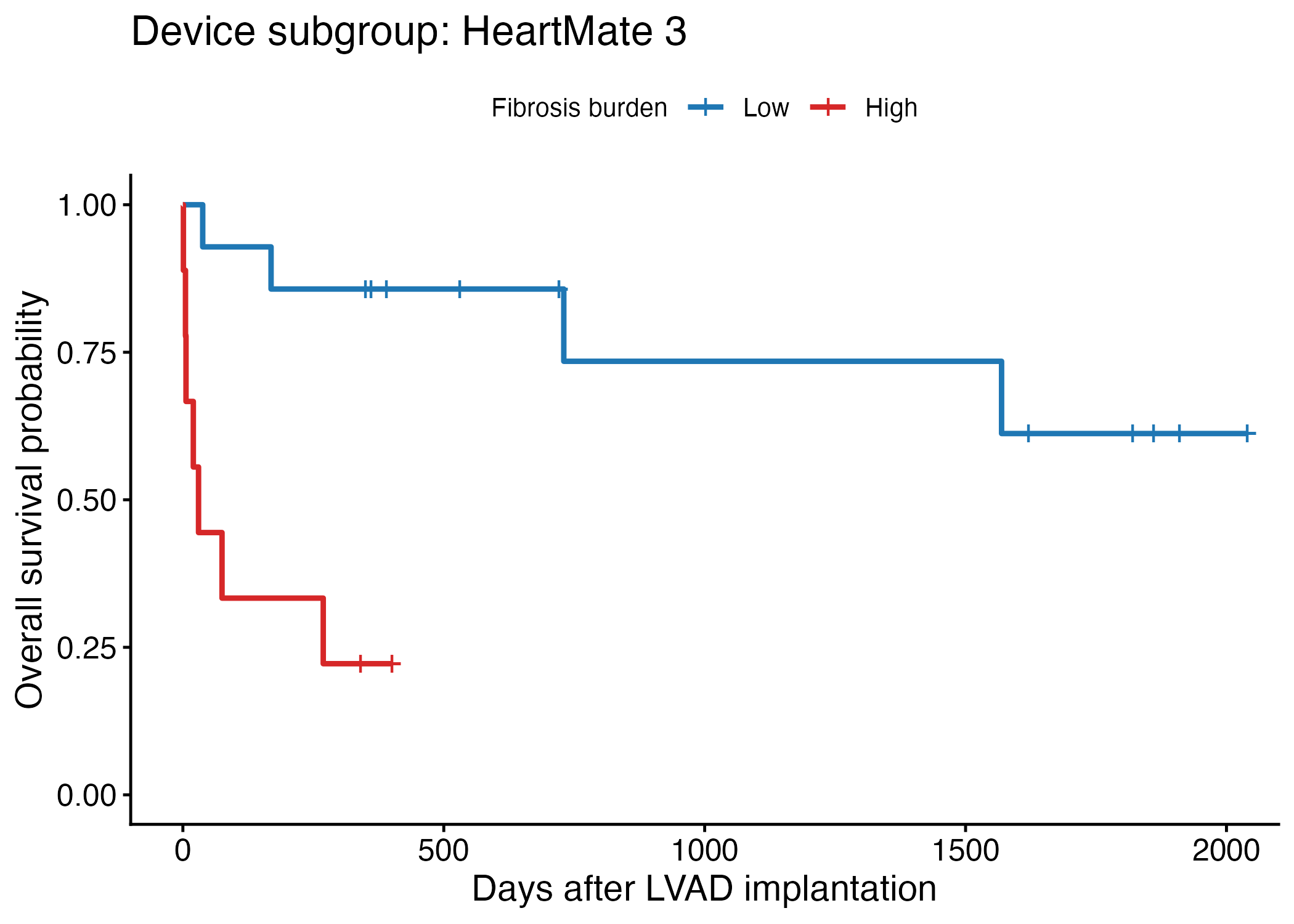

Supplement: xvag135_Supplementary_Data [file xvag135_supplementary_data.zip › Supplementary Fig S1A.png]

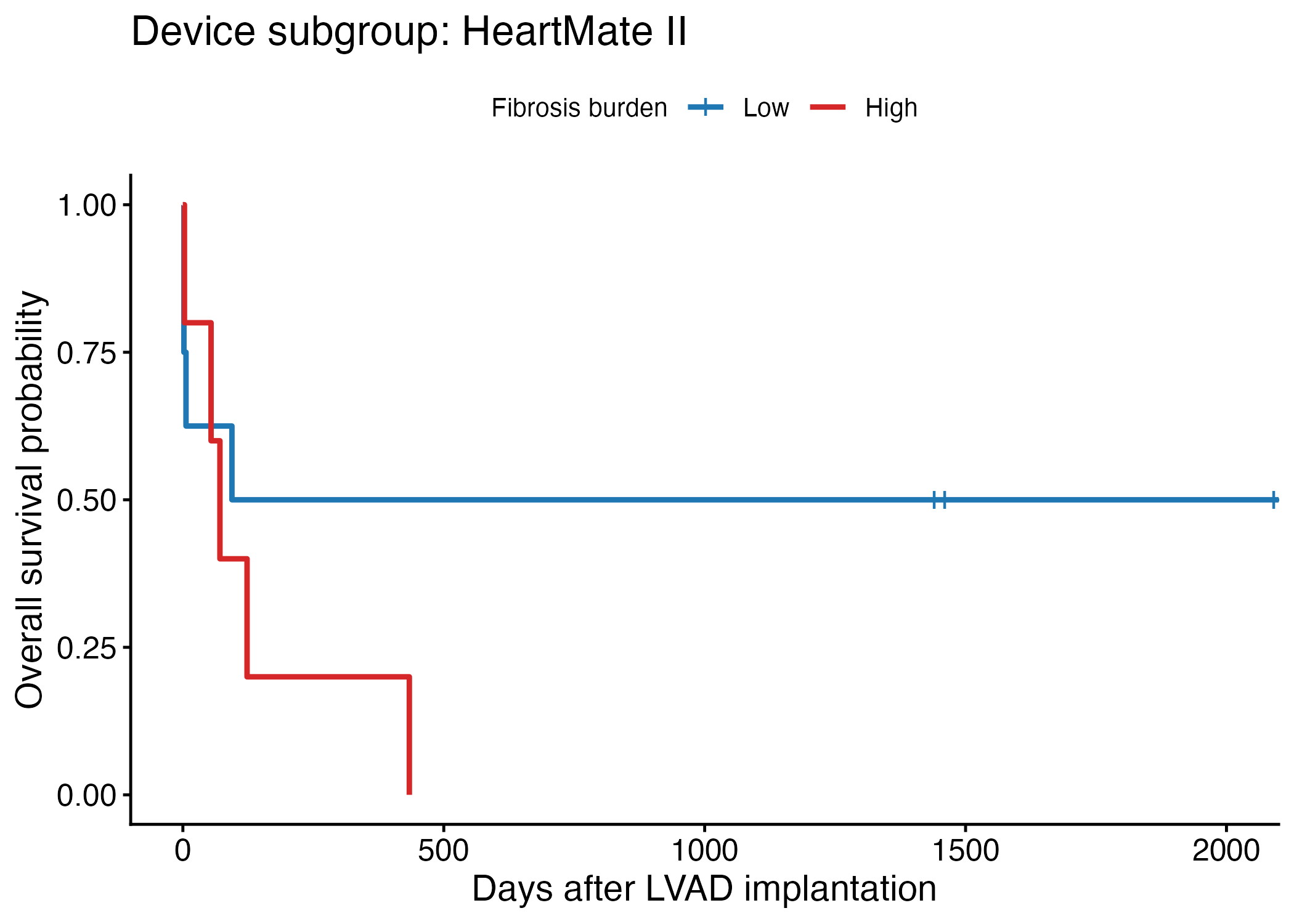

Supplement: xvag135_Supplementary_Data [file xvag135_supplementary_data.zip › Supplementary Fig S1B.png]

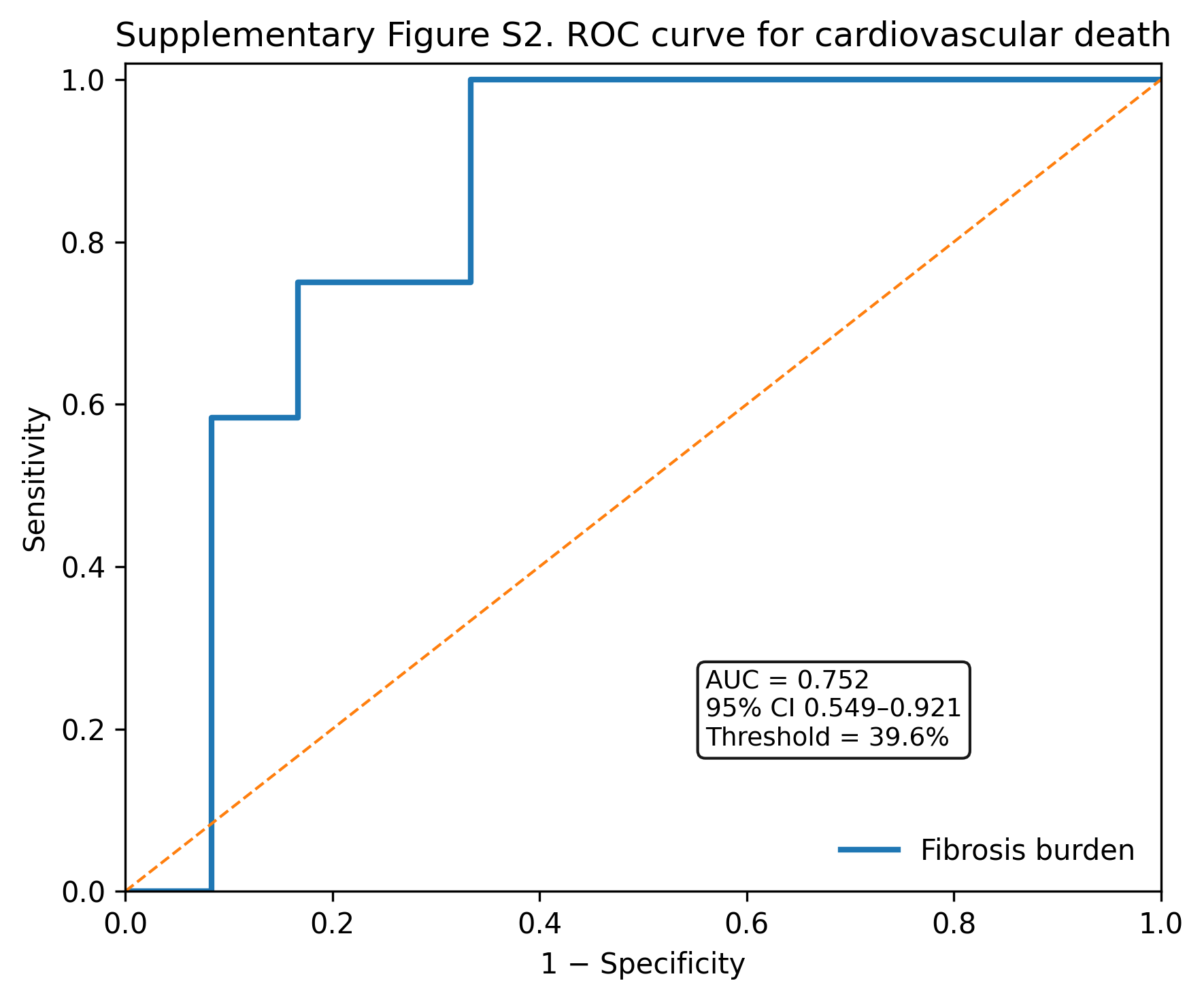

Supplement: xvag135_Supplementary_Data [file xvag135_supplementary_data.zip › Supplementary Figure S2.png]

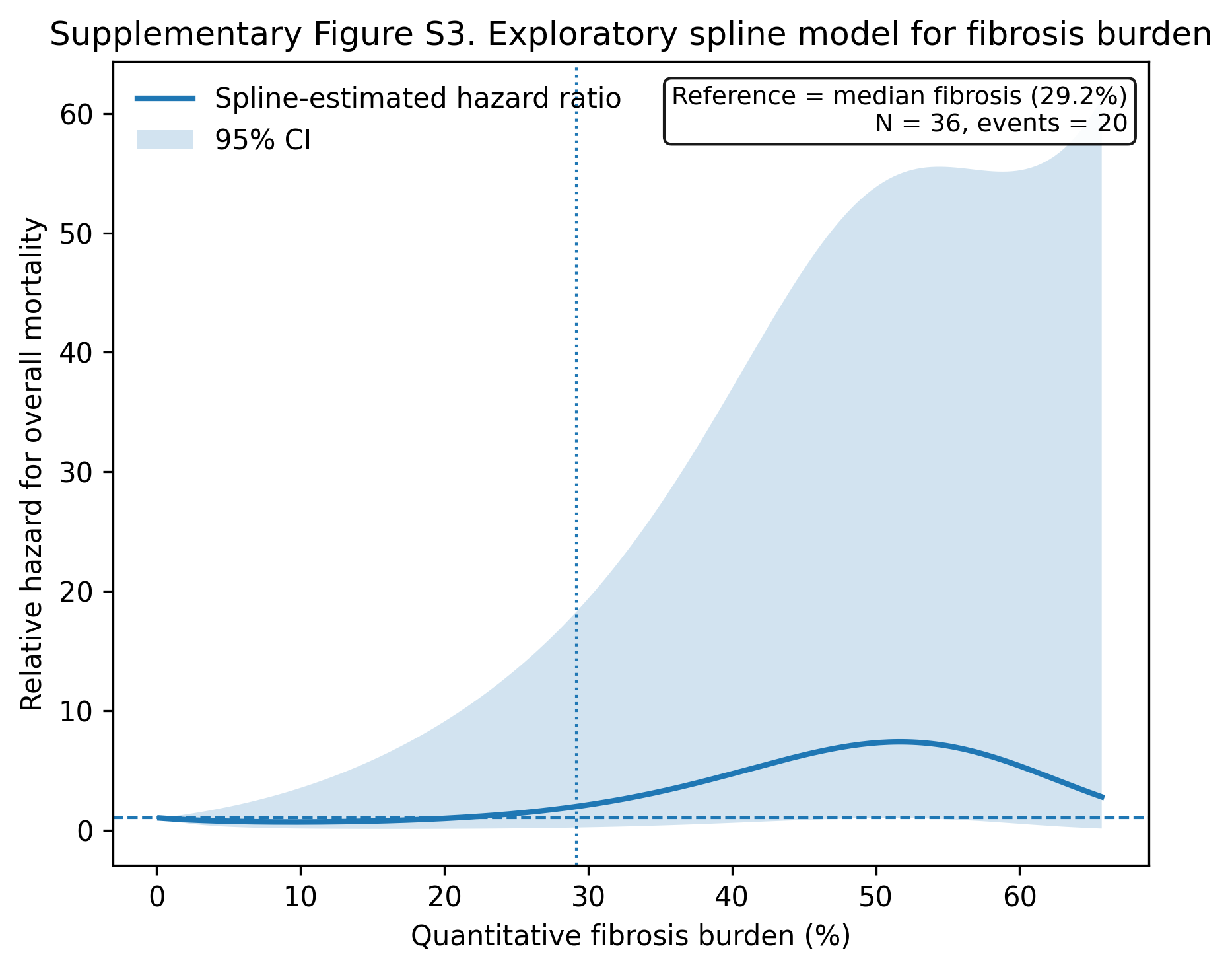

Supplement: xvag135_Supplementary_Data [file xvag135_supplementary_data.zip › Supplementary Figure S3.png]
